# Supplementary material for: Prevalence and correlates of malaria and undernutrition among acutely febrile children visiting temporary malaria screening sites in war-torn areas of Northeast Ethiopia
Source: PLoS One. 2024 Oct 17;19(10):e0311931. doi: 10.1371/journal.pone.0311931 (PMC11486399; doi:10.1371/journal.pone.0311931)
Supplement: S1 File — (PDF) [file pone.0311931.s001.pdf]

Questioners for malaria and under nutrition among children visiting temporal malaria screening sites in the war-torn areas of Northeast Ethiopia

**Socio-demographic characteristics**	
Sex	
Age (years)	
Residence	
Highest level of education of the parents/guardians	
Parents occupation	
Family size	
Testing site	
Malaria result	A. Positive (specify species)<br>B. Negative
**Behavioural and environmental factors for malaria**	
History of malaria	
Stagnant water near to the house	
Proper utilization of ITNs	
Number of ITNs (adequate/inadequate)	
Outdoor stay at night	
Environmental management	
Knowledge of care giver on malaria prevention	
Nutritional status	
**Nutritional status and risk factors**	
Nutritional status	A. Under-nutrition (specify its type)<br>B. Normal
Highest level of education of the parents/guardians	
Family size	
Meal frequency per day	
Dietary diversity score (DDS)	
Presence of diarrhoea/vomiting	
Malaria status (infected/non-infected)	
