# Supplementary material for: Prevalence and correlates of malaria and undernutrition among acutely febrile children visiting temporary malaria screening sites in war-torn areas of Northeast Ethiopia
Source: PLoS One. 2024 Oct 17;19(10):e0311931. doi: 10.1371/journal.pone.0311931 (PMC11486399; doi:10.1371/journal.pone.0311931)
Supplement: Attachment — Submitted filename: PONE-D-24-27373_reviewer.pdf [file pone.0311931.s002.pdf]

PLOS ONE

# Magnitude and Determinants of Malaria and Undernutrition among Acutely Febrile Children Visiting Temporary Malaria Screening Sites in War-Torn Areas of Northeast Ethiopia

--Manuscript Draft--

| | |
|---|---|
| **Manuscript Number:** | PONE-D-24-27373 |
| **Article Type:** | Research Article |
| **Full Title:** | Magnitude and Determinants of Malaria and Undernutrition among Acutely Febrile Children Visiting Temporary Malaria Screening Sites in War-Torn Areas of Northeast Ethiopia |
| **Short Title:** | Magnitude and Determinants of Malaria and Undernutrition among Acutely Febrile Children, Northeast Ethiopia |
| **Corresponding Author:** | Habtu Debash<br>Wollo University<br>Dessie, ETHIOPIA |
| **Keywords:** | Malaria; Under-nutrition; Determinants; Children; War-torn areas; Ethiopia |
| **Abstract:** | Background<br><br>Malaria and malnutrition pose challenges for children in conflict-affected areas. Understanding the prevalence and risk factors for these issues in war-torn communities is important to effectively design aid efforts and select interventions. This study aimed to determine the prevalence and associated factors of malaria and malnutrition among febrile children in Northeast Ethiopia to help address these problems.<br><br>Methods<br><br>A cross-sectional study was conducted from November 2022 to January 2023. Approximately 422 children were enrolled using a systematic random sampling technique. Data on risk factor variables were collected via questionnaire. Capillary blood samples were collected from each child to prepare thick and thin blood films, which were stained with Giemsa and examined microscopically. Height and weight measurements were also taken using a meter and standard calibrated balance. The data were analyzed in SPSS 26.0 using bivariable and multivariable logistic regression to determine associations between determinants, malaria infection, and malnutrition. Statistical significance was set at $p < 0.05$.<br><br>Results<br><br>The overall malaria prevalence among screened children at temporary sites in the conflict-affected areas of Northeast Ethiopia was 65.9% (278/422). Plasmodium falciparum, P. vivax, and mixed infections accounted for 74.1%, 19.8%, and 6.1% of the cases, respectively. The presence of stagnant water, improper utilization of ITN, inadequate number of ITNs per family size and absence of environmental management were independent predictors of malaria. On the other hand, the overall malnutrition prevalence was 54.7% (231/422), including 26.5% underweight, 16.8% stunted, and 11.4% wasted. Dietary diversity score, meal frequency, and confirmed malaria infection were significantly associated with malnutrition.<br><br>Conclusion<br><br>Both malaria and malnutrition burdens were high among the children in this study. The findings suggest combined prevention measures for malaria and malnutrition should be strengthened in this region. |
| **Order of Authors:** | Habtu Debash |
| | Ermiyas Alemayehu |
| | |

Powered by Editorial Manager® and ProduXion Manager® from Aries Systems Corporation

| | |
|---|---|
| | Melaku Ashagrie Belete |
| | Hussien Ebrahim |
| | Ousman Mohammed |
| | Daniel Gebretsadik |
| | Mihret Tilahun |
| | Agumas Shibabaw |
| | Zewudu Mulatie |
| | Bruktawit Eshetu |
| | Saba Gebremichael |
| | Alemu Gedefie |
| **Additional Information:** | |
| **Question** | **Response** |
| **Financial Disclosure**<br><br>Enter a financial disclosure statement that describes the sources of funding for the work included in this submission. Review the submission guidelines for detailed requirements. View published research articles from *PLOS ONE* for specific examples.<br><br>This statement is required for submission and **will appear in the published article** if the submission is accepted. Please make sure it is accurate.<br><br>**Funded studies**<br>Enter a statement with the following details:<br>• Initials of the authors who received each award<br>• Grant numbers awarded to each author<br>• The full name of each funder<br>• URL of each funder website<br>• Did the sponsors or funders play any role in the study design, data collection and analysis, decision to publish, or preparation of the manuscript?<br><br>Did you receive funding for this work? | The author(s) received no specific funding for this work. |
| **Competing Interests**<br><br>Use the instructions below to enter a competing interest statement for this submission. On behalf of all authors, | The authors have declared that no competing interests exist. |

| | |
|---|---|
| disclose any competing interests that could be perceived to bias this work—acknowledging all financial support and any other relevant financial or non-financial competing interests.<br><br>This statement is required for submission and **will appear in the published article** if the submission is accepted. Please make sure it is accurate and that any funding sources listed in your Funding Information later in the submission form are also declared in your Financial Disclosure statement.<br><br>View published research articles from *PLOS ONE* for specific examples.<br><br>**NO authors have competing interests**<br><br>Enter: *The authors have declared that no competing interests exist.*<br><br>**Authors with competing interests**<br><br>Enter competing interest details beginning with this statement:<br><br>*I have read the journal's policy and the authors of this manuscript have the following competing interests: [insert competing interests here]*<br><br>* typeset | |
| **Ethics Statement**<br><br>Enter an ethics statement for this submission. This statement is required if the study involved:<br><br>• Human participants<br>• Human specimens or tissue<br>• Vertebrate animals or cephalopods<br>• Vertebrate embryos or tissues<br>• Field research<br><br>Write "N/A" if the submission does not require an ethics statement. | Ethical clearance was obtained from the Ethical Review Committee of the College of Medicine and Health Sciences at Wollo University on the date 21/10/2022 with protocol number of CMHS/126/2022. Permission was also obtained from the Waghemra Zone Health Office. After briefly describing the significance of the study to the children's parents or guardians, informed written consent was obtained from them. Confidentiality of the data was maintained. Finally, participants who were infected with the Plasmodium parasite received antimalarial treatment according to national malaria treatment guidelines. Those with undernutrition were referred to Tefera Hailu Memorial Hospital for further investigation. All methods were carried out in accordance with the Declaration of Helsinki guidelines and regulations. |

| | |
|---|---|
| General guidance is provided below. Consult the submission guidelines for detailed instructions. **Make sure that all information entered here is included in the Methods section of the manuscript.**<br><br>**Format for specific study types**<br><br>**Human Subject Research (involving human participants and/or tissue)**<br>• Give the name of the institutional review board or ethics committee that approved the study<br>• Include the approval number and/or a statement indicating approval of this research<br>• Indicate the form of consent obtained (written/oral) or the reason that consent was not obtained (e.g. the data were analyzed anonymously)<br><br>**Animal Research (involving vertebrate animals, embryos or tissues)**<br>• Provide the name of the Institutional Animal Care and Use Committee (IACUC) or other relevant ethics board that reviewed the study protocol, and indicate whether they approved this research or granted a formal waiver of ethical approval<br>• Include an approval number if one was obtained<br>• If the study involved *non-human primates*, add *additional details* about animal welfare and steps taken to ameliorate suffering<br>• If anesthesia, euthanasia, or any kind of animal sacrifice is part of the study, include briefly which substances and/or methods were applied<br><br>**Field Research**<br><br>Include the following details if this study involves the collection of plant, animal, or other materials from a natural setting:<br>• Field permit number<br>• Name of the institution or relevant body that granted permission | |
| **Data Availability**<br><br>Authors are required to make all data | Yes - all data are fully available without restriction |

*Powered by Editorial Manager® and ProduXion Manager® from Aries Systems Corporation*

| | |
|---|---|
| underlying the findings described fully available, without restriction, and from the time of publication. PLOS allows rare exceptions to address legal and ethical concerns. See the PLOS Data Policy and FAQ for detailed information.<br><br>A Data Availability Statement describing where the data can be found is required at submission. Your answers to this question constitute the Data Availability Statement and **will be published in the article**, if accepted.<br><br>**Important:** Stating 'data available on request from the author' is not sufficient. If your data are only available upon request, select 'No' for the first question and explain your exceptional situation in the text box.<br><br>Do the authors confirm that all data underlying the findings described in their manuscript are fully available without restriction? | |
| **Describe where the data may be found in full sentences. If you are copying our sample text, replace any instances of XXX with the appropriate details.**<br><br>• If the data are **held or will be held in a public repository**, include URLs, accession numbers or DOIs. If this information will only be available after acceptance, indicate this by ticking the box below. For example: *All XXX files are available from the XXX database (accession number(s) XXX, XXX.*).<br>• If the data are all contained **within the manuscript and/or Supporting Information files**, enter the following: *All relevant data are within the manuscript and its Supporting Information files.*<br>• If neither of these applies but you are able to provide **details of access elsewhere**, with or without limitations, please do so. For example:<br><br>*Data cannot be shared publicly because of [XXX]. Data are available from the* | All data generated in this study are included in the manuscript. The data sets analysed during the current study are available from the corresponding author on reasonable request. |

| | |
|---|---|
| *XXX Institutional Data Access / Ethics Committee (contact via XXX) for researchers who meet the criteria for access to confidential data.*<br><br>*The data underlying the results presented in the study are available from (include the name of the third party and contact information or URL).*<br>• This text is appropriate if the data are owned by a third party and authors do not have permission to share the data.<br><br>* typeset | |
| Additional data availability information: | |

Powered by Editorial Manager® and ProduXion Manager® from Aries Systems Corporation

Click here to access/download;Manuscript;Revised malaria and undernutrition manuscript .docx

# Magnitude and Determinants of Malaria and Undernutrition among Acutely Febrile Children Visiting Temporary Malaria Screening Sites in War-Torn Areas of Northeast Ethiopia

Habtu Debash[1*], Ermiyas Alemayehu[1], Melaku Ashagrie Belete[1], Hussien Ebrahim[1], Ousman Mohammed[1], Daniel Gebretsadik[1], Mihret Tilahun[1], Agumas Shibabaw[1], Zewudu Mulatie[1], Bruktawit Eshetu[1], Saba Gebremichael[1], Alemu Gedefie[1]

[1]Department of Medical Laboratory Sciences, College of Medicine and Health Sciences, Wollo University, Dessie, Ethiopia

***Corresponding author:**

**Mr. Habtu Debash**

E-mail address: habtudebash@gmail.com (HD)

Phone: +251921264072

**Co-authors Email:**

ermiyas0009@gmail.com (EA)

melakuashagrie@gmail.com (MAB)

husshosam@gmail.com (HE)

ousmanabum@gmail.com (OM)

gebretsadikd@gmail.com (DG)

tilahunmihret21@gmail.com (MT)

agumas2000@gmail.com (AS)

zewudumulatie@gmail.com (ZM)

bruktieshetu1989@gmail.com (BE)

sabagmichal2@gmail.com (SG)

alemugedefie@gmail.com (AG)

## Abstract

**Background:** Malaria and malnutrition pose challenges for children in conflict-affected areas. Understanding the prevalence and risk factors for these issues in war-torn communities is important to effectively design aid efforts and select interventions. This study aimed to determine the prevalence and associated factors of malaria and malnutrition among febrile children in Northeast Ethiopia to help address these problems.

**Methods:** A cross-sectional study was conducted from November 2022 to January 2023. Approximately 422 children were enrolled using a systematic random sampling technique. Data on risk factor variables were collected via questionnaire. Capillary blood samples were collected from each child to prepare thick and thin blood films, which were stained with Giemsa and examined microscopically. Height and weight measurements were also taken using a meter and standard calibrated balance. The data were analyzed in SPSS 26.0 using bivariable and multivariable logistic regression to determine associations between determinants, malaria infection, and malnutrition. Statistical significance was set at $p < 0.05$.

**Results:** The overall malaria prevalence among screened children at temporary sites in the conflict-affected areas of Northeast Ethiopia was 65.9% (278/422). *Plasmodium falciparum*, *P. vivax*, and mixed infections accounted for 74.1%, 19.8%, and 6.1% of the cases, respectively. The presence of stagnant water, improper utilization of ITN, inadequate number of ITNs per family size and absence of environmental management were independent predictors of malaria. On the other hand, the overall malnutrition prevalence was 54.7% (231/422), including 26.5% underweight, 16.8% stunted, and 11.4% wasted. Dietary diversity score, meal frequency, and confirmed malaria infection were significantly associated with malnutrition.

**Conclusion**: Both malaria and malnutrition burdens were high among the children in this study. The findings suggest combined prevention measures for malaria and malnutrition should be strengthened in this region.

**Keywords**: Malaria, Under-nutrition, Determinants, Children, War-torn areas, Ethiopia

## Introduction

Both natural phenomena and human activities impact the emergence of diseases. Significant human influences include armed conflicts like wars as well as regional tribal and sectarian struggles. This leads to destroy infrastructure and cause healthcare systems to collapse, indirectly affecting disease transmission [1]. A year of conflict in Ethiopia left thousands dead, forced more than two million people from their homes, and pushed parts of the country into famine. More than 542,000 people have been displaced as a result of domestic conflict in Ethiopia's Amhara region. The Waghemra zone was severely impacted by the region's internal turmoil [2]. Armed conflicts have been shown to negatively influence the availability and accessibility of healthcare. The war resulted in widespread devastation of healthcare

institutions, putting additional strain on the already precarious healthcare system in the impacted areas. The negative consequences include the destruction of health facilities, the murder of health workers, malnutrition with serious consequences for children, an increase in cases of mental and emotional disorders, epidemics, and other public health issues [3].

Due to the conflict, anti-malarial activities that had previously kept malaria under control were halted. However, if the health system fails, anti-malarial treatments are interrupted, or non-immune people migrate, an epidemic is possible in high-transmission areas [4]. The demise of the program led to the re-emergence of malaria, transforming the territory into a malaria-endemic area [5]. This may potentially contribute to the spread of medication resistance, jeopardizing long-term elimination goals [6]. The lack of protective immunity, increased concentration of people in exposed settings, limited availability of insecticide-treated bed nets (ITNs), inadequate indoor residual spraying (IRS), and insufficient rapid clinical diagnostic and treatment responses make conflict areas prone to malaria infections [7, 8].

Other risk factors include nighttime activities outside, wearing shorts, living in unfinished buildings, inadequate drainage, and acute malnutrition in children [9, 10]. However, combating malnutrition is difficult in places of violent conflict, as women and children are disproportionately affected. The increased global hunger was mostly caused by conflicts, explaining why conflict zones bore the burden of food insecurity and malnutrition [11-13]. Conflicts negatively influence food security by causing large displacements, deep economic recessions, rising inflation and unemployment, and eroding funds for social safety and healthcare. Conflicts disrupt food production, harvesting, processing, transportation, and marketing in areas where agriculture is the primary source of income, resulting in low resilience. As a result, worsening food insecurity might heighten tensions and risks of additional conflicts [11, 12].

People in these circumstances are at higher risk of malnutrition and its consequences [7]. Some research [14, 15] has documented socio-demographic characteristics such as age, gender, education level, and occupation are factors that influence nutrition. Moreover, low birth weight, immunization status, poor maternal nutritional status, food intake have all been linked to childhood malnutrition [16, 17]. Malaria infection and nutritional health have a complicated relationship. Previous research indicates that malaria may increase the incidence and severity of malnutrition, and that malnutrition may raise the risk of malaria infection

[18]. The region has faced over a year of ongoing internal conflict, which has seriously weakened the healthcare system and disrupted the delivery of crucial services. As a result, communities in Northeast Ethiopia are particularly vulnerable to public health issues like malaria and malnutrition.

Understanding the magnitude and key risk factors for malaria infections and under nutrition in war-affected communities is critical for designing humanitarian responses and selecting appropriate intervention strategies for under nutrition and malaria prevention, control, and even elimination. This is only possible with adequate research and evidence to support the development of effective and sustainable management strategies. The purpose of this study was to provide an overview of the prevalence of malaria infections, under nutrition, and local context risk factors among acute febrile children seeking health care at temp[illegible]l malaria screening sites in war-torn areas of Northeast Ethiopia.

**Methods**

**Study area, design and period**

A descriptive cross-sectional survey was conducted on acutely febrile children receiving healthcare at temporary malaria screening sites from November 1st, 2022 to January 30th, 2023 in Waghemra Zone, Northeast Ethiopia. Waghemra Zone is one of eleven zones in Ethiopia's Amhara Region. The Waghemra zone is defined by the following latitude and longitude coordinates: 12° 45' 54" N, 38° 50' 34.8"E. The study site is located in the dissected landscapes of Waghemra Zone's Tekezie Basin, where natural and man-made calamities such as violence, migration, and food insecurity have posed severe challenges since 2021/22. This zone is divided into eight districts with a total population of 536,129 people. This sero-survey was conducted in Abergelle Woreda where children came for treatment at temporary malaria screening and treatment sites. However, some kebeles in the Aber[illegible] District were excluded from the data collection due to the ongoing war in the area. In addition to the conflict, the climate and terrain of the region provide suitable breeding grounds for Anopheles mosquitoes.

## Sample size determination

The sample size was calculated using a single population proportion formula, by taking 95% confidence limits ($Z\alpha/2 = 1.96$), and 5% margin of error (d) with a maximum proportion of 50%. So, the sample size = $Z(\alpha/2)^2\ P\ (1- P)/\ d^2$, $(1.96)\ )^2\ \ast\ 0.221\ (1 – 0.221)/\ (0.05)\ )^2\ =$

384. Considering a 10% non-response rate, a total of 422 children were ultimately included in the study.

## Sampling techniques and procedure

A stratified sampling method was used to select the representative sample size. The Waghemra Zone health department had established rapid malaria response teams that provide malaria diagnosis and treatment at sites in Saka, Debrebirhan, and Beltarf. The total number of malaria-diagnosed febrile patients in the two months prior to the study was used as the sampling frame for each diagnostic and treatment center. Based on this, the total sample size was proportionally allocated such that 212, 124, and 86 febrile children were included from the Saka, Debrebirhan, and Beltarf sites respectively. Systematic random sampling was applied to select study participants from each center.

## Inclusion and exclusion criteria

Children who complained of a fever ≥37.5°C and were clinically suspected of having malaria, who were referred to the laboratory for a blood film examination. Children parents who are agreed to participate were included in the study. Critically ill children and individuals who had taken anti-malarial drugs within 4 weeks before data collection were excluded.

## Data collection methods

### Socio-demographic and other factors

To collect information from each child, a pre-tested questionnaire was used, which included related elements such as background characteristics, behaviors, environmental, and other factors important for malaria infection and under-nutrition (Supplementary file 1). Before final data collection, the tool was developed in English and translated into the local language. Parents or guardians of the children were interviewed about socio-demographic parameters and other determinant factors. These factors include insecticide-treated net (ITN) conditions and availability, ITN utilization and number, IRS, presence of stagnant water, nighttime outdoor stays, and past malaria health information.

**Blood sample collection, processing, and examination**.

Following the interview, blood samples were aseptically collected from each child using a disposable sterile lancet via a finger prick. Approximately 6 µl and 2 µl of blood were used to prepare thick and thin smears, respectively. Both thin and thick blood films were prepared

and air dried. The thin blood film was then fixed with methanol and allowed to air dry. Thin and thick blood films were stained with 10% Giemsa staining solution for 10 minutes. After being rinsed with distilled water, the slides were air dried. Finally, the dried slides were examined by laboratory technologists at the health facilities. Thick smears were used to detect *Plasmodium* infections, while thin smears were used to identify parasite species using an oil immersion objective.

**Anthropometric measurements**

Clinical nurses measured each child's weight and height using a digital scale that measures to the nearest 0.1 kilograms and a vertical meter that measures to the nearest 0.1 cm, respectively. Before measurement, the participant removed their shoes, jacket, and hair clips and positioned their feet together flat on the ground, with heels touching the back plate of the measuring instrument, legs straight, buttocks against the backboard, scapula against the backboard, and arms at their sides. The study participants' weight and height were measured twice by separate nurses to decrease subjective error, and the mean measurement result was reported. The WHO AnthroPlus software was used to estimate malnutrition among schoolchildren. The WHO AnthroPlus software was used to calculate z-scores of height-for-age (HAZ) and body mass index-for-age z-score (BAZ). Children with a z-score < -2SD were classified as underweight (WHZ < -2SD), stunted (HAZ < -2SD) and wasted (BAZ < -2SD) [19].

**Dietary diversity score:** Dietary consumption in the 24 hours prior to the survey was assessed using questionnaires developed based on Food and Agriculture Organization (FAO) guidelines [20]. Consumed foods were classified into 10 food groups [21]. Consumption from each food group was reported, and one point was given if the food was consumed at least once in the last 24 hours and zero points if not consumed within that period. Therefore, the dietary diversity score (DDS) ranged from 0 to 10, and participants with a DDS of ≤3 were classified as poor diversity whereas participants with a DDS of 4–6 and >6 were classified as middle and high diversity respectively [21].

## Quality control

The data collectors underwent training and the questionnaire was pre-tested. To ensure the quality and integrity of the Giemsa staining, positive and negative blood film samples were taken. The microscopic slides were reviewed individually by two qualified laboratory technicians. Before being concluded as negative, hundreds of microscopic fields of the thick smear were inspected. Any disparities between the findings of the first and second technicians were resolved by a third senior laboratory technologist.

## Statistical analysis

The gathered information was entered into Epi Data Version 4.2 and then exported to SPSS Version 26 for analysis. Narration and tabulation are used to present the descriptive statistics. A variable with a P-value of less than or equal to 0.25 in the bivariable analysis was selected as a potential confounding variable for the multivariable logistic regression analysis. To determine significantly associated variables for the risk of malaria infection and undernutrition, a P-value of less than 0.05 was used.

**Ethical approval and consent to participate**

Ethical clearance was obtained from the Ethical Review Committee of the College of Medicine and Health Sciences at Wollo University on the date 21/10/2022 with protocol number of CMHS/126/2022. Permission was also obtained from the Waghemra Zone Health Office. After briefly describing the significance of the study to the children's parents or guardians, informed written consent was obtained from them. Confidentiality of the data was maintained. Finally, participants who were infected with the *Plasmodium* parasite received antimalarial treatment according to national malaria treatment guidelines. Those with undernutrition were referred to Tefera Hailu Memorial Hospital for further investigation. All methods were carried out in accordance with the Declaration of Helsinki guidelines and regulations.

# Results

## Socio-demographic characteristics of study participants

A total of 422 children participated in the study, with a 100% response rate. Of these, 238 (56.4%) were females and 184 (43.6%) were males. In terms of age, 114 (27.0%) were less than or equal to 5 years old, 132 (31.3%) were between 6 to 10 years, and 176 (41.7%) were between 11 to 15 years. The majority of the children came from rural areas (89.1%), while the rest came from urban areas. The higher proportion (68.2%) of the children were from

households with five or more family members, and 53.8% had never received a formal education and hence could not read or write. The Saka testing site had the highest proportion of participants (50.2%), while Beltarf had the lowest (20.4%). Of the overall study participants, the vast majority (83.4%) were children of parents who were farmers (Table 1).

**Table 1:** Socio-demographic characteristics of children visiting temporal malaria screening sites in the war-torn areas of Northeast Ethiopia, 2023

| **Variables** | **Alternatives** | **Number (%)** |
|---|---|---|
| Sex | Male | 238 (56.4) |
| | Female | 184 (43.6) |
| Age (years) | ≤5 | 114 (27.0) |
| | 6-10 | 132 (31.3) |
| | 11-15 | 176 (41.7) |
| Residence | Rural | 376 (89.1) |
| | Urban | 46 (10.9) |
| Highest level of education of the parents/guardians | Unable to write and read | 227 (53.8) |
| | Elementary school | 119 (28.2) |
| | High school | 48 (11.4) |
| | Higher education | 28 (6.6) |
| Parents occupation | Government employee | 18 (4.3) |
| | Merchant | 52 (12.3) |
| | Farmer | 352 (83.4) |
| Family size | ⩾5 | 288 (68.2) |
| | <5 | 134 (21.8) |
| Testing site | Saka | 212 (50.2) |
| | Debre brihan | 124 (29.4) |
| | Beltarf | 86 (20.4) |

## *Plasmodium* Parasite Distribution with Socio-demographic Variables

The overall prevalence of malaria in this study was 65.9% (278/422) (95% CI: 61.0-70.3). Of these cases, malaria prevalence was 156 (56.1%) in males and 122 (43.9%) in females. The difference in malaria prevalence by sex was not statistically significant (P = 0.871). Among the *Plasmodium* parasite-infected children, 70 (61.4%) were in the less than 5 years age group, 98 (74.2%) were between 6 and 10 years, and 110 (62.5%) were between 11 and 15

years. Younger children had a lower prevalence of the *Plasmodium* parasite, with a statistically significant difference (P = 0.049). There was a higher prevalence of malaria parasites in children from rural residents (251; 66.8%) compared to children from urban residents (58.7%), but the difference was not statistically significant (P = 0.276) (Table 2).

**Table 2:** Socio-demographic characteristics and malaria infection of children visiting temporal malaria screening sites in the war-torn areas of Northeast Ethiopia, 2023

| **Variables** | **Alternatives** | **Total tested** | **Malaria** | | |
|---|---|---|---|---|---|
| | | | **Positive** | **Negative** | **P-value** |
| | | **Number (%)** | **Number (%)** | **Number (%)** | |
| Sex | Male | 238 (56.4) | 156 (65.5) | 82 (34.5) | 0.871 |
| | Female | 184 (43.6) | 122 (66.3) | 62 (33.7) | |
| Age (years) | ≤5 | 114 (27.0) | 70 (61.4) | 44 (38.6) | 0.049 |
| | 6-10 | 132 (31.3) | 98 (74.2) | 34 (25.8) | |
| | 11-15 | 176 (41.7) | 110 (62.5) | 66 (37.5) | |
| Residence | Rural | 376 (89.1) | 251 (66.8) | 125 (33.2) | 0.276 |
| | Urban | 46 (10.9) | 27 (58.7) | 19 (41.7) | |
| Highest level of education of the parents/guardians | Unable to write and read | 227 (53.8) | 150 (66.1) | 77 (33.9) | 0.469 |
| | Elementary school | 119 (28.2) | 74 (62.2) | 45 (37.8) | |
| | High school | 48 (11.4) | 36 (75.0) | 12 (25.0) | |
| | Higher education | 28 (6.6) | 18 (64.3) | 10 (35.7) | |
| Occupation | Government employee | 18 (4.3) | 13 (72.2) | 5 (27.8) | |
| | Merchant | 52 (12.3) | 39 (75.0) | 13 (25.0) | |
| | Farmer | 352 (83.4) | 226 (64.2) | 126 (35.8) | 0.261 |
| Family size | ⩾5 | 288 (68.2) | 194 (67.4) | 94 (32.6) | 0.346 |
| | <5 | 134 (21.8) | 84 (62.7) | 50 (37.3) | |

## *Plasmodium* species distribution by testing sites

In this study, there was a higher prevalence of malaria parasites at the Saka testing site (78.3%) compared to the D/brihan (72.8%) and Beltarf (62.2%) testing sites. The difference in prevalence between the different testing sites was statistically significant (P = 0.006) (Table 2). Among the malaria positive cases, the dominant *Plasmodium* species was P. falciparum in 206 cases (74.1%), followed by P. vivax in 55 cases (19.8%), and the remaining 17 cases (6.1%) were mixed infections of *P. falciparum* and *P. vivax* (Table 3).

**Table 3**: The relative proportion of *Plasmodium* species among confirmed cases by testing sites in the war-torn areas of Northeast Ethiopia, 2023

| Testing sites | Total Bf examined, n (%) | Positive n (%) | *P. falciparum* n (%) | *P. vivax* n (%) | Mixed n (%) | Negative n (%) | P-value |
|---|---|---|---|---|---|---|---|
| Saka | 212 (50.2) | 152 (71.7) | 119 (78.3) | 25 (16.4) | 8 (5.3) | 60 (28.3) | 0.006 |
| D/brihan | 124 (29.4) | 81 (65.3) | 59 (72.8) | 18 (22.2) | 4 (4.9) | 43 (34.7) | |
| Beltarf | 86 (20.4) | 45 (52.3) | 28 (62.2) | 12 (26.7) | 5 (11.1) | 41 (47.7) | |
| **Total** | 422 (100.0) | 278 (65.9) | 206 (74.1) | 55 (19.8) | 17 (6.1) | 144 (34.1) | |

## Factors associated with malaria infection

The presence of stagnant water, improper utilization of ITNs, an inadequate number of ITNs per family size, and the absence of environmental management were independent predictors of malaria. Participants who lived near mosquito breeding sites were more likely to have positive test results for malaria infections (adjusted odds ratio (AOR = 3.225; 95% CI: 1.987–5.234) compared to those who lived farther from mosquito breeding sites. Children who did not use ITNs were 3.7 times more likely to be infected with the *Plasmodium* parasite than those who did use ITNs (AOR = 3.701, 95% CI = 2.233–6.135). Likewise, the odds of contracting malaria among respondents who lived in households with an inadequate number of ITNs per family size were about three times higher than their counterparts (AOR: 3.561; 95% CI: 2.089–6.070). Furthermore, those who did not control their living environment were three times more likely to be at risk of contracting malaria (AOR = 3.315, 95% CI: 1.900–5.784) (Table 4).

**Table 4**: Bivariable and multivariable regression analysis of determinant factors for malaria in the war-torn areas of Northeast Ethiopia, 2023

| **Variables** | **Alternatives** | **Malaria status** | | **COR (95%CI)** | **P-value** | **AOR (95%CI)** | **P-value** |
|---|---|---|---|---|---|---|---|
| | | **Positive n (%)** | **Negative n (%)** | | | | |
| Sex | Male | 156 (65.5) | 82 (34.5) | 0.967(.644-1.451) | 0.871 | | |
| | Female | 122 (66.3) | 62 (33.7) | 1 | | | |
| Age | ≤5 | 70 (61.4) | 44 (38.6) | 0.955(0.588-1.550) | 0.851 | | |
| | 6-10 | 98 (74.2) | 34 (25.8) | 0.552(0.321-0.950) | 0.302 | | |
| | 11-15 | 110 (62.5) | 66 (37.5) | 1 | | | |
| Residence | Rural | 251 (66.8) | 125 (33.2) | 0.708(379-1.322) | 0.278 | | |
| | Urban | 27 (58.7) | 19 (41.7) | 1 | | | |
| History of malaria | Yes | 99 (69.7) | 43 (30.3) | 1.299(.842-2.003) | 0.236* | 1.008(0.579-1.754) | 0.978 |
| | No | 179 (63.9) | 101 (36.1) | 1 | | | |
| Stagnant water near to the house | Yes | 188 (80.3) | 46 (19.7) | 4.450(2.892-6.849) | 0.001* | 3.225(1.987-5.234) | 0.001* |
| | No | 90 (47.9) | 98 (52.1) | 1 | | | |
| Proper utilization of ITNs | Yes | 90 (48.9) | 94 (51.1) | 1 | | | |
| | No | 188 (79.0) | 50 (21.0) | 3.927(2.567-6.008) | 0.001* | 3.701(2.233-6.135) | 0.001* |
| Number of ITNs | Adequate | 49 (43.4) | 64 (56.6) | | | | |
| | Inadequate | 229 (74.1) | 80 (25.9) | 3.739(2.382-5.868) | 0.001* | 3.561(2.089-6.070) | 0.001* |
| Outdoor stay at night | Yes | 62 (81.6) | 14 (18.4) | 2.665(1.435-4.952) | 0.002* | 1.082(0.492-2.380) | 0.844 |
| | No | 216 (62.4) | 130 (37.6) | 1 | | | |
| Environmental management | Yes | 42 (43.3) | 55 (56.7) | 1 | | | |
| | No | 236 (72.6) | 89 (27.4) | 3.472(2.171-5.555) | 0.0001* | 3.315(1.900-5.784) | 0.001* |
| Knowledge of care giver on malaria prevention | Good | 87 (54.4) | 73 (45.6) | 1 | | | |
| | Poor | 191 (72.9) | 71 (27.1) | 2.257(1.493-3.413) | 0.0001* | 1.859(0.739-3.034) | 0.482 |
| Nutritional status | Under nutrition | 170 (73.6) | 61 (26.4) | 2.142(1.423-3.225) | 0.0001* | 2.020(0.843-3.283) | 0.310 |
| | Normal | 108 (56.5) | 83 (43.5) | 1 | | 1 | |

*Significantly associated variables, AOR: Adjusted Odd Ratio, COR: Cru[illegible]dd Ratio

## Nutritional status and associated factors

The overall prevalence of undernutrition among the children was 54.7% (231/422) (50.3-59.1). Of this, the prevalences of underweight, stunting, and wasting were 26.5% (22.3-30.9), 16.8% (13.3-20.6), and 11.4% (8.2-14.5), respectively (Table 5).

**Table 5:** The prevalence of under nutrition among children in the war-torn areas of Northeast Ethiopia, 2023

| **Nutritional status** | **Categories** | | **Frequency (n)** | **Percentage (%)** |
|---|---|---|---|---|
| | Under nutrition | Wasted | 48 | 11.4 |
| | | Stunted | 71 | 16.8 |
| | | Underweight | 112 | 26.5 |
| | | Overall | 231 | 54.7% |
| | Normal | | 191 | 45.3% |

In the current study, the majority (72.7%) of the undernourished children were from families with five or more members. Similarly, 130 (56.3%) cases had caregivers who did not have any formal education. However, neither family size (P = 1.43) nor the caregiver's educational status (P = 0.356) were significantly associated with malnutrition. The binary logistic regression model showed that study participants who had a dietary diversity score (DDS) ≤ 3 (AOR = 2.147, 95% CI: 1.374–3.355), meal frequency of at most 3 times per day (AOR = 7.484, 95% CI: 4.740–11.817), and confirmed malaria infection (AOR = 2.215, 95% CI: 1.387–3.540) were strongly associated with undernutrition (Table 6).

**Table 6:** Binary logistic regression analysis of potential risk factors associated with under nutrition among children in the war-torn areas of Northeast Ethiopia, 2023

| **Variables** | **Categories** | **Under nutrition** | | COR (95% CI) | P-value | AOR(95% CI) | P-value |
|---|---|---|---|---|---|---|---|
| | | **Yes** | **No** | | | | |
| | | Number (%) | Number (%) | | | | |
| Age (years) | ≤5 | 63 (55.3) | 51 (44.7) | 1.353(0.843-2.171) | 0.282 | | |
| | 6-10 | 84 (63.6) | 48 (36.4) | 0.706(0.423-1.178) | 0.260 | | |
| | 11-15 | 84 (47.7) | 92 (52.3) | 1 | | | |
| Residence | Rural | 203 (54.0) | 173 (46.0) | 0.754(0.403-1.411) | 0.377 | | |
| | Urban | 28 (60.9) | 18 (39.1) | 1 | | | |
| Highest level of education of the parents/guardians | Unable to write and read | 130 (57.3) | 97 (42.7) | 0.536(0.227-1.268) | 0.356 | | |
| | Elementary school | 57 (47.9) | 62 (52.1) | 1.340(0.718-2.501) | 0.458 | | |
| | High school | 24 (50.0) | 24 (50.0) | 1.458(0.934-2.276) | 0.297 | | |
| | Higher education | 20 (71.4) | 8 (28.6) | 1 | | | |
| Family size | ⩾5 | 168 (58.3) | 120 (41.7) | 1.578(1.045-2.383) | 0.03* | 1.426(0.887-2.292) | 1.43 |
| | <5 | 63 (47.0) | 71 (53.0) | 1 | | 1 | |
| Meal frequency per day | ≤3 | 154 (79.4) | 40 (20.6) | 7.55(4.848-11.759) | 0.001* | 7.484(4.740-11.817) | 0.001* |
| | >3 | 77 (33.8) | 151 (66.2) | 1 | | | |
| Dietary diversity score (DDS) | ≤3 | 137 (63.1) | 80 (36.9) | 2.022(1.370-2.985) | 0.001* | 2.147(1.374-3.355) | 0.001* |
| | >3 | 94 (45.9) | 111 (54.1) | 1 | | 1 | |
| Presence of diarrhoea/vomiting | Yes | 144 (59.5) | 98 (40.5) | 1.571(1.065-2.318) | 0.023* | 1.339(0.853-2.100) | 0.205 |
| | No | 87 (48.3) | 93 (51.7) | 1 | | 1 | |
| Malaria | Infected | 170 (61.2) | 108 (38.8) | 2.142(1.423-3.225) | 0.001* | 2.215(1.387-3.540) | 0.001* |
| | Not infected | 61 (42.4) | 83 (57.6) | 1 | | 1 | |

*Significantly associated variables, AOR: Adjusted Odd Ratio, COR: Crud Odd Ratio

## Discussion

This present study showed that malaria is a public health problem among acutely febrile children visiting temporary malaria screening sites in the war-torn areas of northeast Ethiopia. The study revealed that the overall malaria prevalence was 65.9% (95% CI: 61.0–70.3). This finding was consistent with a prior study in Ethiopia, where the prevalence of malaria was 66.4% in villages surrounding Lake Langano in the Oromia region of Ethiopia [22] and in Nigeria (77.6%) [23].

In contrast, this result was higher than studies that found a 14.7% prevalence in the South Gondar Zone of northwest Ethiopia [24], 43.8% in the Jawi district of northwest Ethiopia [25], 20.5% in the East Shewa Zone of Ethiopia [26], 22.1% in Arba Minch Zuria District in southern Ethiopia [27], 58.2% in Anambra State, Nigeria [28], 55.3% in Niger [18], and 36.3% in Tanzania [29]. The high prevalence of malaria in this study might be related to the internal conflict in the study area over the past two years. This has resulted in deterioration of the health system and interruption of malaria prevention measures that previously kept malaria under control. This difference may also be due to seasonal variation as our study was conducted during a major malaria transmission season, contrary to the other studies.

This study revealed that the predominant *Plasmodium* species detected in the blood of the children was *P. falciparum* in 206 cases (74.1%), followed by *P. vivax* in 55 cases (19.8%), and the remaining 17 cases (6.1%) showed mixed infections. This result disagreed with previous studies done in Ethiopia [24-27] and the national *Plasmodium* species prevalence, which indicated *P. falciparum* and *P. vivax* accounted for 60 and 40% of the malaria cases in the country, respectively. The higher dominance of *P. falciparum* over *P. vivax* is related to *P. falciparum*'s high proliferation in the host cell, the parasite's ability to infect all ages of red blood cells, and the parasite's treatment resistance [30]. Moreover, 6.1% of the children had mixed infections with *P. falciparum* and *P. vivax*. The reason for the mixed infections may be due to simultaneous infection by both species.

Malaria infections are frequently complicated and multi-factorial, influenced by both natural and man-made causes [31]. Those children living within a 1 km radius of a mosquito breeding site were three times as likely to develop malaria compared to those living mosquito free breeding sites. Stagnant water created by heavy rains provides an ideal breeding environment for mosquitoes and contributes to malaria epidemics [32, 33]. A similar

conclusion was reached in research conducted in Simada district of northwest Ethiopia [33], and Waghemra Zone of northeast Ethiopia [34], which found an association between living near such water sources and contracting malaria.

This study also found that households that did not regularly use ITNs were 3.701 times more likely than those who used them regularly to be infected with the malaria parasite. This result is supported by similar previous studies conducted in the South Gondar and Arba Minch Zuria districts of Ethiopia [24, 27]. Insecticide-treated nets are an effective vector control method for preventing malaria transmission. When they are used consistently, the risk of getting a mosquito bite might be decreased [35, 36]. As a result, in malaria-endemic areas, local authorities should enforce the regular use of ITNs.

Children who lived in households with an inadequate number of ITNs were 3.561 times more likely to develop malaria. Inadequate ITNs in households might enforce infrequent utilization of ITNs in the community. It is recommended that there be at least one insecticide-treated net (ITN) for every two persons in the household. Furthermore, those children living in poorly controlled environments were three times at higher risk of contracting the disease. This finding is supported by a report from the Simada District in northwest Ethiopia [33]. Hence, there should be sustainable, integrated, and coordinated malaria prevention and control measures and proper water management to eliminate mosquito breeding sites [37].

On the other hand, undernutrition is one of the most serious health issues facing children in poor countries like Ethiopia, and it can have a negative impact on their physical and mental development [38, 39]. In the studied area, 54.7% (95% CI: 50.3-59.1) of children suffered from malnutrition. This result was higher than studies reported from Sekota Town in northeast Ethiopia (46.3%) [40], rural Ethiopia (48.5%) [41], and the pooled prevalence of under nutrition among under-five-year-old children in Ethiopia (49.0%) [42]. In armed internal conflict, individuals cannot be commit fully to jobs due to lack of safety, which affects families' ability to buy food [43], a key determinant of malnutrition [44]. Healthcare services are frequently in short supply during conflicts, either because medical facilities are inaccessible due to insecurity or because they become potential targets as part of war strategy [45]. Routine immunization, a major determinant of malnutrition, is one of the most affected healthcare services for children during wars [44].

According to the findings of this study, underweight (26.5%) was far more common than stunting or wasting. The prevalence of underweight was consistent with a study done in

Debre Berhan Town, Ethiopia (26%) [46]. In this study, the prevalence of stunting was 16.8%. This finding was higher than studies conducted in Mecha, northwest Ethiopia (11.6%) [39]. However, the finding was lower than previous studies conducted in Debre Berhan Town, Ethiopia (41%) [46] and Northern Samar, the Philippines (49.2%) [47].

Furthermore, 11.4% of the study participants were wasted. This was consistent with studies done in Mecha, northwest Ethiopia (10.8%) [39] and Sekota town, northeast Ethiopia (10.2%) [51]. However, this finding was lower than the finding from northeast Ethiopia (20.7%) [48], Debre Berhan Town, Ethiopia (41%) [46] and the Philippines (59.7%) [47]. This difference might be due to differences in community awareness about nutrition, the socioeconomic status of families, health service coverage, levels of poverty, the prevalence of diarrheal disease, and dietary diversity.

Dietary diversity score, meal frequency, and having a confirmed malaria infection were independent predictors of under-nutrition among children in this study. A similar association was reported between dietary diversity score in Sekota Town, Northeast Ethiopia [40] and Burkina Faso [49], and meal frequency in Dessie Town and Bahir Dar, Ethiopia [48, 50] which showed a statistically significant association. Food and nutrition insecurity becomes increasingly worse in areas affected by armed conflict. Children living in war-torn settings face a disproportionate burden of malnutrition and poor health outcomes. Therefore, there is a need for a stronger evidence-based humanitarian response to crises.

Additionally, being infected with malaria was a predictor of undernutrition, which agrees with a study conducted in Bahir Dar, Ethiopia [51]. Malaria infections can impair food and nutrient absorption by reducing appetite, increasing metabolic demands, weakening the transport of nutrients to tissues, and altering the gut lumen, leading to undernutrition [52, 53]. At a population level, there is evidence that an increase in malaria infection rates was associated with peaks in admission to therapeutic feeding programs. Meanwhile, children with malnutrition also showed a greater risk of complications from malaria requiring hospitalization [54].

## Conclusion and recommendations

Both malaria and undernutrition become increasingly worse in areas affected by armed conflict. The prevalence of both malaria and undernutrition was high among children in the war-torn areas of Northeast Ethiopia. Malaria infection was significantly associated with the

presence of stagnant water in residential areas, improper utilization of ITNs, an inadequate number of ITNs, and the absence of environmental management. On the other hand, dietary diversity score, meal frequency, and having a confirmed malaria infection were independent predictors of undernutrition. The results imply the need for strengthening integrated strategies to reduce both malaria and undernutrition. With the prolonged political instability in Ethiopia, sustainable malaria and nutritional interventions are necessary to alleviate difficult conditions in conflict-exposed areas. Improved access to all malaria and undernutrition interventions is critical for achieving the Sustainable Development Goals, but consistent progress across different sectors is also required.

## Declarations

### Data availability

All relevant data are included in the published article.

### Conflicts of interest

Authors declare that they have no conflict of interest.

### Consent for publication

Not applicable.


### Funding

The research project was not funded by any organization.


### Authors' contributions

Habtu Debash, Ermiyas Alemayehu, Melaku Ashagrie Belete and Hussien Ebrahim conceived and designed the study, prepared the proposal, supervised data collection, analyzed, and interpreted the data. Habtu Debash, Ousman Mohammed, Daniel Gebretsadik, Mihret Tilahun and Alemu Gedefie had participated in data collection, data analysis, and interpretation of the result, collecting scientific literature, critical appraisal of articles for inclusion, analysis, and interpretation of the findings. Habtu Debash drafted and prepared the manuscript for publication. Ermiyas Alemayehu, Melaku Ashagrie Belete, Hussien Ebrahim and Alemu Gedefie critically reviewed the manuscript. All the authors have read and approved the final version of the manuscript and agreed to be accountable for all aspects of the work.

### Data Availability

All data generated in this study are included in the manuscript. The data sets analysed during the current study are available from the corresponding author on reasonable request.

**Competing interests**

The author(s) declared no potential conflicts of interest with respect to the research, authorship, and/or publication of this article.

**Funding**

The author(s) received no financial support for the research, authorship, and/or publication of this article.

**Acknowledgements**

The authors would like to thank Wollo University for giving ethical clearance and providing necessary materials. The authors also thank the study participants, data collectors, and Waghemra Zone Health Office staffs for their support and unreserved cooperation in making this study to be a fruitful work.

## References

1. Morse, S., *Factors and determinants of disease emergence.* Revue scientifique et technique-Office international des épizooties, 2004. **23**(2): p. 443-452.
2. Abebe, H. and G. Belaineh, *Key Considerations: Social Science Perspectives for Emergency Response to the Conflict in Northern Ethiopia.* 2022.
3. Oladayo, N.A., *The impact of conflict on health outcomes: a systematic evidence from sub-saharan Africa.* Mgbakoigba: Journal of African Studies, 2019. **8**(1): p. 88-100.
4. Idris, I.O., et al., *Factors influencing severity of recurrent malaria in a conflict-affected state of South Sudan: an unmatched case-control study.* Conflict and Health, 2022. **16**(1): p. 1-10.
5. Kakar, Q., M. Khan, and K. Bile, *Malaria control in Pakistan: new tools at hand but challenging epidemiological realities.* EMHJ-Eastern Mediterranean Health Journal, 16 (Supp.), 54-60, 2010, 2010.
6. Gari, T. and B. Lindtjørn, *Reshaping the vector control strategy for malaria elimination in Ethiopia in the context of current evidence and new tools: opportunities and challenges.* Malaria journal, 2018. **17**(1): p. 1-8.
7. Sherrard-Smith, E., et al., *Systematic review of indoor residual spray efficacy and effectiveness against Plasmodium falciparum in Africa.* Nature communications, 2018. **9**(1): p. 4982.
8. Costantini, C., et al., *Reply to Wagman et al.: Data-driven assessments should establish the landscape of what is "within reach" of malaria transmission control.* Proceedings of the National Academy of Sciences, 2022. **119**(38): p. e2211931119.
9. Takarinda, K.P., et al., *Factors associated with a malaria outbreak at Tongogara refugee camp in Chipinge District, Zimbabwe, 2021: a case–control study.* Malaria Journal, 2022. **21**(1): p. 94.
10. Tepa, A., et al., *Nutritional Status and Humoral Immune Response to Plasmodium falciparum in Children Aged 6–59 Months.* Journal of Tropical Medicine, 2020. **2020**: p. 1-7.

11. FAO, W., *Monitoring food security in countries with conflict situations: A joint FAO/WFP update for the United Nations Security Council.* New York: Food and Agriculture Organization of the United Nations, World Food Programme, 2018.
12. Kiarie, J., et al., *The prevalence and associated factors of undernutrition among under-five children in South Sudan using the standardized monitoring and assessment of relief and transitions (SMART) methodology.* BMC nutrition, 2021. **7**(1): p. 1-11.
13. Crises, G.N.A.F. and F. Network, *Global Report on Food Crises: Joint analysis for better decisions.* Rome:(2020).[Google Scholar], 2020.
14. Zeray, A., G.D. Kibret, and C.T. Leshargie, *Prevalence and associated factors of undernutrition among under-five children from model and non-model households in east Gojjam zone, Northwest Ethiopia: a comparative cross-sectional study.* BMC nutrition, 2019. **5**: p. 1-10.
15. Díez-Navarro, A., et al., *Female eco-stability and severe malnutrition in children: Evidence from humanitarian aid interventions of Action Against Hunger in African, Asian and Latin American countries.* Nutricion Clinica y Dietetica Hospitalaria, 2017. **34**: p. 127-34.
16. Medhin, G., et al., *Prevalence and predictors of undernutrition among infants aged six and twelve months in Butajira, Ethiopia: the P-MaMiE Birth Cohort.* BMC public health, 2010. **10**: p. 1-15.
17. Darsene, H., et al., *Magnitude and predictors of undernutrition among children aged six to fifty nine months in Ethiopia: a cross sectional study.* Archives of public health, 2017. **75**: p. 1-11.
18. Oldenburg, C.E., et al., *Malaria and nutritional status among children with severe acute malnutrition in Niger: a prospective cohort study.* Clinical Infectious Diseases, 2018. **67**(7): p. 1027-1034.
19. Pimenta, F.S., et al., *Agreement between subjective global nutritional assessment and the nutritional assessment of the World Health Organization.* Jornal de Pediatria, 2018. **94**: p. 602-608.
20. Liese, A., *Explaining varying degrees of openness in the food and agriculture organization of the United Nations (FAO).* Transnational actors in global governance: Patterns, explanations and implications, 2010: p. 88-109.
21. Kennedy, G., T. Ballard, and M.C. Dop, *Guidelines for measuring household and individual dietary diversity*. 2011: Food and Agriculture Organization of the United Nations.
22. Mulugeta, B., *Prevalence of Malaria and Utilization of ITN in Children Under Five.* Oromia Regional State, Ethiopia, 2013: p. 46-47.
23. Igwe, N.M., et al., *Prevalence and parasite density of asymptomatic malaria parasitemia among unbooked paturients at Abakaliki, Nigeria.* Journal of Basic and Clinical Reproductive Sciences, 2014. **3**(1): p. 44-48.
24. Workineh, L., et al., *Prevalence of malaria and associated factors among children attending health institutions at South Gondar Zone, Northwest Ethiopia: a cross-sectional study.* Global Pediatric Health, 2021. **8**: p. 2333794X211059107.
25. Hailu, T., et al., *Incidence of Plasmodium infections and determinant factors among febrile children in a district of Northwest Ethiopia; a cross-sectional study.* Tropical Diseases, Travel Medicine and Vaccines, 2018. **4**(1): p. 1-6.
26. Haji, Y., A.W. Fogarty, and W. Deressa, *Prevalence and associated factors of malaria among febrile children in Ethiopia: a cross-sectional health facility-based study.* Acta Tropica, 2016. **155**: p. 63-70.
27. Abossie, A., et al., *Prevalence of malaria and associated risk factors among febrile children under five years: a cross-sectional study in Arba Minch Zuria district, South Ethiopia.* Infection and drug resistance, 2020: p. 363-372.
28. Nwaorgu, O. and B. Orajaka, *Prevalence of malaria among children 1–10 years old in communities in awka north local government area, Anambra State South East Nigeria.* African Research Review, 2011. **5**(5): p. 264-281.

29. Sumari, D., et al., *Malaria prevalence in asymptomatic and symptomatic children in Kiwangwa, Bagamoyo district, Tanzania.* Malaria journal, 2017. **16**(1): p. 1-7.
30. Assefa, A. *The third Ethiopian Malaria Indicator Survey 2015 (EMIS-2015)*. in *28th Annual conference, 2016*. 2016.
31. Defi, G.B., et al., *A malaria outbreak in Ameya Woreda, South-West Shoa, Oromia, Ethiopia, 2012: weaknesses in disease control, important risk factors.* American Journal of Health Research, 2015. **3**(3): p. 125-129.
32. Debela, M.B., A.B. Kahsay, and T.M. Mokonnon, *Malaria outbreak and contracting factors in Afar region, Ethiopia, 2016.* Journal of Public Health and Epidemiology, 2018. **10**(7): p. 233-240.
33. Workineh, B., et al., *Malaria outbreak investigation and contracting factors in Simada District, Northwest Ethiopia: a case–control study.* BMC research notes, 2019. **12**: p. 1-6.
34. Debash, H., et al., *Malaria surveillance, outbreak investigation, response and its determinant factors in Waghemra Zone, Northeast Ethiopia: unmatched case–control study.* Scientific Reports, 2023. **13**(1): p. 9938.
35. Karunamoorthi, K., *Vector control: a cornerstone in the malaria elimination campaign.* Clinical Microbiology and Infection, 2011. **17**(11): p. 1608-1616.
36. Debash, H., et al., *Prevalence and associated risk factors of malaria among febrile under-five children visiting health facilities in Ziquala district, Northeast Ethiopia: A multicenter cross-sectional study.* Plos one, 2022. **17**(10): p. e0276899.
37. Keiser, J., et al., *Effect of irrigation and large dams on the burden of malaria on a global and regional scale.* American Journal of Tropical Medicine and Hygiene, 2005. **72**(4): p. 392-406.
38. Bain, L.E., et al., *Malnutrition in Sub–Saharan Africa: burden, causes and prospects.* Pan African Medical Journal, 2013. **15**(1).
39. Tewabe, T. and A. Belachew, *Determinants of nutritional status in school-aged children in Mecha, Northwest Ethiopia.* Current Therapeutic Research, 2020. **93**: p. 100598.
40. Debash, H., M. Alemu, and H. Bisetegn, *The prevalence of intestinal parasites, undernutrition and their associated risk factors among school-age children in Sekota Town, Northeast Ethiopia: A community-based cross-sectional study.* Health Science Reports, 2023. **6**(3): p. e1137.
41. Endris, N., H. Asefa, and L. Dube, *Prevalence of malnutrition and associated factors among children in rural Ethiopia.* BioMed research international, 2017. **2017**.
42. Kassie, G.W. and D.L. Workie, *Determinants of under-nutrition among children under five years of age in Ethiopia.* BMC Public Health, 2020. **20**(1): p. 1-11.
43. Kadir, A., S. Shenoda, and J. Goldhagen, *Effects of armed conflict on child health and development: a systematic review.* PloS one, 2019. **14**(1): p. e0210071.
44. Pridmore, P. and R. Carr-Hill, *Tackling the drivers of child undernutrition in developing countries: what works and how should interventions be designed?* Public health nutrition, 2011. **14**(4): p. 688-693.
45. Footer, K.H. and L.S. Rubenstein, *A human rights approach to health care in conflict.* International Review of the Red Cross, 2013. **95**(889): p. 167-187.
46. Menalu, M.M., et al., *Assessment of prevalence and factors associated with malnutrition among under-five children in Debre Berhan town, Ethiopia.* International Journal of General Medicine, 2021: p. 1683-1697.
47. Papier, K., et al., *Childhood malnutrition and parasitic helminth interactions.* Clinical Infectious Diseases, 2014. **59**(2): p. 234-243.
48. Bisetegn, H., et al., *Prevalence and Determinant Factors of Intestinal Parasitic Infections and Undernutrition among Primary School Children in North-Central Ethiopia: A School-Based Cross-Sectional Study.* Journal of Parasitology Research, 2023. **2023**.
49. Sié, A., et al., *Dietary diversity and nutritional status among children in rural Burkina Faso.* International health, 2018. **10**(3): p. 157-162.

50. Hailegebriel, T., *Undernutrition, intestinal parasitic infection and associated risk factors among selected primary school children in Bahir Dar, Ethiopia.* BMC infectious diseases, 2018. **18**: p. 1-11.
51. Yimam, H., *Assessment of the Relationship between Under-Nutrition and Malaria in Preschool Children at Bahir Dar Special Zone, Ethiopia: A Comparative Cross-Sectional Study*. 2014, Addis Ababa University.
52. Katona, P. and J. Katona-Apte, *The interaction between nutrition and infection.* Clinical Infectious Diseases, 2008. **46**(10): p. 1582-1588.
53. Stephensen, C.B., *Burden of infection on growth failure.* The Journal of nutrition, 1999. **129**(2): p. 534S-538S.
54. Wurr, C., J. Zeydner, and S. van der Kam, *Malnutrition peaks during malaria epidemic in Northwest Nigeria.* Field Exchange 50, 2015: p. 6.

Click here to access/download
**Supporting Information**
Ethical clearance for malaria and undernutrition_3.pdf

Click here to access/download
**Supporting Information**
Point by pont response to edditors comment.pdf

Questionaire

Click here to access/download

**Supporting Information**

Questionnaire.pdf
