# Supplementary material for: Prevalence and correlates of malaria and undernutrition among acutely febrile children visiting temporary malaria screening sites in war-torn areas of Northeast Ethiopia
Source: PLoS One. 2024 Oct 17;19(10):e0311931. doi: 10.1371/journal.pone.0311931 (PMC11486399; doi:10.1371/journal.pone.0311931)
Supplement: Attachment — Submitted filename: Point by point response.pdf [file pone.0311931.s005.pdf]

Point by point response to reviewers' and editors' comments

Dear editor and reviewers.

Thank you very much for your positive feedback on our work. We greatly appreciate you taking the time to review our manuscript. Based on the comments raised by the reviewers, we revised the manuscript and indicated all changes made in a tracked changes file. Your acknowledgment that we have adequately addressed all points raised through our revisions is encouraging. Thank you again for your feedback and consideration of our manuscript for potential publication.

Manuscript Title: Magnitude and Determinants of Malaria and Undernutrition among Acutely Febrile Children Visiting Temporary Malaria Screening Sites in War-Torn Areas of Northeast Ethiopia

Response to reviewer #1

Comment #1: The authors have thoroughly responded to all of the feedback from both reviewers. I have no further comments on the manuscript.

Response: Thank you for taking the time to evaluate our manuscript and provide feedback. We appreciate you acknowledging that we have addressed all the points raised by the reviewer. Your recommendation that the manuscript requires no further revision is helpful and an important step before a decision on publication can be made. Your review has strengthened our work by confirming that we have adequately responded to the comments and suggestions for improvement. We believe the manuscript is now suitably revised for consideration for publication. Thank you again for your thoughtful review.

Response to reviewer #2

Reviewer #2: Comments to the Author:

Congratulations on thoroughly addressing all the previous suggestions and comments. The revised manuscript is significantly improved in terms of clarity and methodological precision. Below are my final comments:

Comment Suggestions:

The comparison with other studies has been expanded and now includes clearer justifications for the observed differences in prevalence, which enriches the discussion.
The section on study limitations has been improved, offering detailed insights into potential biases, especially regarding the exclusion of certain areas due to conflict.
I suggest a final grammatical revision to ensure the fluency of the text is impeccable.

Overall, all my previous concerns have been satisfactorily addressed, and I see no further obstacles to accepting this manuscript for publication.

Response: Thank you for your thorough review and helpful feedback on our revised manuscript. We are glad to hear that you feel we have satisfactorily addressed your previous concerns. We appreciate you noting that the expansion of the comparison with other studies has strengthened the discussion by including clearer justifications for differences observed. Additionally, your comments regarding our improved section on study limitations and the provision of detailed insights into potential biases are greatly valued.

As per your suggestion, we have conducted a final grammatical revision to ensure textual fluency is impeccable. We believe the manuscript is now rigorous and well-rounded as a result of incorporating the changes recommended by you and the other reviewer. Thank you for confirming there are no further obstacles to accepting our work for publication. We feel privileged to contribute our research to the journal under your guidance. Thank you again.
