# Supplementary material for: Prevalence and correlates of malaria and undernutrition among acutely febrile children visiting temporary malaria screening sites in war-torn areas of Northeast Ethiopia
Source: PLoS One. 2024 Oct 17;19(10):e0311931. doi: 10.1371/journal.pone.0311931 (PMC11486399; doi:10.1371/journal.pone.0311931)
Supplement: Attachment — Submitted filename: Review Paper Malaria in Etiopia Plos One .docx [file pone.0311931.s003.docx]

### Comments and Suggestions for Manuscript Revision

**Manuscript Number: PONE-D-24-27373**

**Manuscript Title: Magnitude and Determinants of Malaria and Undernutrition among Acutely Febrile Children Visiting Temporary Malaria Screening Sites in War-Torn Areas of Northeast Ethiopia**

#### Introduction

**Justification and contextualization:** The introduction clearly presents the relevance of the study, highlighting the importance of understanding the prevalence and risk factors of malaria and undernutrition in conflict areas.

#### Methodology

**Study design:** The study is cross-sectional, conducted between November 2022 and January 2023. The use of systematic random sampling is appropriate for this type of study.

**Data collection:** The data collection via questionnaire, capillary blood samples, and anthropometric measurements is well described. However, it would be useful to include information about the validation of the questionnaire used.

**Statistical analysis:** The use of bivariate and multivariate logistic regression is appropriate. However, it is essential to ensure that all confounding variables are adequately controlled.

- **Suggestion:** Include a detailed description of the statistical analysis, including the variable selection strategy (stepwise) and how the adjustment of the multivariate models was performed, including the magnitude of the effect after the exponentiation of the beta from the logistic regression model.

#### Results

**Prevalence of malaria and undernutrition:** The results show a high prevalence of malaria (65.9%) and undernutrition (54.7%). It is important to discuss whether these results are consistent with other studies in similar contexts.

- **Suggestion 1:** Simplify the description of the results to avoid excessive repetition and make the reading more fluid. It is not necessary to repeat results for all categories and all numbers that are in the table.
- **Suggestion 2:** Consolidate Table 3 into Table 2 to avoid redundancy and facilitate reading.
- **Suggestion 3:** The column for the total tested in Table 2 is redundant to Table 1. I recommend removing it, as this information is already included in Table 1.

2. **Analysis of risk factors:** The independent factors associated with malaria include the presence of stagnant water, inadequate use of mosquito nets, and lack of environmental management. For undernutrition, dietary diversity and meal frequency are highlighted as significant factors.

#### Discussion

**Comparison with other studies:** The comparison with other studies is made but could be expanded to include more details on why certain prevalences are higher or lower compared to other regions.

- **Suggestion:** Include an explanation of why diarrhea lost significant association after adjustment in the multivariate model and cite relevant studies, as it is a significant predictor in undernutrition. I suggest citing the article "Predictors of nutritional recovery time in children aged 6–59 months with severe acute malnutrition in Sofala Province, Mozambique: survival analysis approach" by Audêncio Victor et al., published in the Journal of Public Health. This study can be accessed at: <https://doi.org/10.1093/pubmed/fdae049>.

**Implications of the results:** The discussion addresses well the implications of the findings for public health. However, it would be useful to include more concrete suggestions on how interventions can be implemented in conflict areas.

**Study limitations:** The limitations are mentioned but could be discussed in more detail, especially regarding potential selection biases and data collection limitations.

#### Conclusion

**Summarize the main findings:** The conclusion adequately summarizes the main findings of the study and suggests the need for integrated strategies to combat malaria and undernutrition in conflict regions.

#### Structure and Clarity

**Manuscript Organization:** The manuscript is well-organized, but the introduction could be more concise.

**Language and Grammar:** The language is clear, but additional grammatical revision may be needed to ensure fluency.

#### Ethical Considerations

**Ethical Approval:** Obtaining ethical approval and informed consent is well documented, which is essential for publication.

#### Recommendations for the Authors

**Improvement in Methodology Description:** Include more details about the validation of the questionnaire and control of confounding variables.

**Deeper discussion of limitations:** Expand the limitations section to include more details about possible biases and limitations of the study.

**Expansion of discussion on interventions:** Provide more concrete and detailed suggestions on how interventions can be implemented and evaluated in conflict contexts.
