# Supplementary material for: Prevalence and correlates of malaria and undernutrition among acutely febrile children visiting temporary malaria screening sites in war-torn areas of Northeast Ethiopia
Source: PLoS One. 2024 Oct 17;19(10):e0311931. doi: 10.1371/journal.pone.0311931 (PMC11486399; doi:10.1371/journal.pone.0311931)
Supplement: Attachment — Submitted filename: Point point responses to editor and reviewer comments .pdf [file pone.0311931.s004.pdf]

Point by point response to reviewers' and editors' comments

Dear Editor and reviewers

Thank you very much for your positive feedback to our work. We revised the manuscript based on comments raised and corrections we made indicated in track change file. Thank you.

Manuscript Title: Magnitude and Determinants of Malaria and Undernutrition among Acutely Febrile Children Visiting Temporary Malaria Screening Sites in War-Torn Areas of Northeast Ethiopia

Response to reviewer #1 comments

Abstract

1. 422---This is not approximate. It is the actual number of children.

Response: thank you, we corrected it.

2. Where were they randomly selected from? Can the data be considered representative of any area / population?

Response: Febrile children were selected using a systematic random sampling technique from febrile children attending temporary malaria screening sites in Abergelie district. Regarding to the representation of the area/population we indicated in the revised manuscript. Thank you.

3. Stadiometer?

   Response: stadiometer was not available in the settings, so, we used meter to measure heights of children.

4. The word "determinants" is misused here and elsewhere in the manuscript. It is not a determinant until there is a statistically significant association and, in a cross-sectional study, I would still be hesitant to call it a determinant even with that. It could be called a correlate or a factor.

   Response: Thank you for your constructive comment, we amend it as correlates.

5. You would generally include data here, at the very least P values. At least indicate directionality.

   Response: we included P-values.

Introduction

6. or it may be that the citation at the end of the paragraph needs to be moved up

   Response: we indicated reference 3.

7. You use "malnutrition" elsewhere and sometimes "under-nutrition." I recommend sticking with undernutrition throughout and treating it as one word.

   Response: we corrected it and consistently used the word undernutrition.

8. Temporal?

   Response: we replaced by temporary. Thank you.

Methods

9. Abergelle vs Abergele?

   Response: Abergele is the correct one, so we correct it.

10. In inclusion and exclusion criteria; presented with?, children's, critical ill-How was this defined?

    Response: we amended and corrected them.

11. Anthropometric measurement; The software is used to derive anthropometric indices. You use the cut-offs to calculate indicators of undernutrition.

    Response: The software calculates anthropometric indices and uses established cut-offs to identify indicators of undernutrition effectively.

12. Errors in table 1, Wasn't this by design? If so, is it needed in the table?

    Response: we made changes. Thank you.

13. Are these proportions different? It seems like it's the column proportion in the "total BF examed" but the rest are rows? It's confusing. Need to include the abbreviation for Bf

    Response: we merged and amend table 2 and 3, made changes, the the relative proportion of Plasmodium species from the total cases expressed in their rows.

14. Same comment as for the p-values. There is no need for three digits after the decimal.

    Response: we corrected them accordingly.

15. This is an unusual way to present undernutrition. It also seems strange that there are no underweight stunted children based on the way that wasted + stunted + underweight add precisely to 231.

    Response: Thank you for your insightful feedback regarding the presentation of undernutrition in our study. The categorization of undernutrition in our study follows WHO definitions, which include underweight, stunting, and wasting. The absence of overlapping undernutrition children may seem unusual, but it highlights that not all stunted children are underweight. This can occur due to various factors affecting growth and weight differently. In fact, a child can be stunted due to chronic malnutrition without being underweight at a given point in time. Thank you.

    Discussion

16. All of the studies cited in these two paragraphs comparable? It seems like you would expected a higher prevalence of malaria when you are specifically measuring it in children presenting with malaria-like symptoms at malaria surveillance sites. If these studies did not have a comparable design, I do not think that it adds anything to the discussion to compare your findings with these from other sites.

    Response: thank you so much we delete it.

17. It is unclear why data from the Philippines are cited here. It's not relevant. Those small-area surveys from within Ethiopia may be relevant, or something from the DHS.

    Response: we amend this paragraph and correct using relevant literatures. Thank you.

Response to reviewer #2 comments

1. **Data collection:** The data collection via questionnaire, capillary blood samples, and anthropometric measurements is well described. However, it would be useful to include information about the validation of the questionnaire used.

   Response: we included information about the validation of the questionnaire we used in the revised manuscript.

2. **Statistical analysis:** The use of bivariate and multivariate logistic regression is appropriate. However, it is essential to ensure that all confounding variables are adequately controlled. **Suggestion:** Include a detailed description of the statistical analysis, including the variable selection strategy (stepwise) and how the adjustment of the multivariate models was performed, including the magnitude of the effect after the exponentiation of the beta from the logistic regression model.
3. **Response:** we incorporated the comments in this revised manuscript. Thank you.

Results

4. **Prevalence of malaria and undernutrition:** The results show a high prevalence of malaria (65.9%) and undernutrition (54.7%). It is important to discuss whether these results are consistent with other studies in similar contexts.

- **Suggestion 1:** Simplify the description of the results to avoid excessive repetition and make the reading more fluid. It is not necessary to repeat results for all categories and all numbers that are in the table.

- **Response:** Based on this comment, we have minimized the description of tables to avoid excessive repetition.
- **Suggestion 2:** Consolidate Table 3 into Table 2 to avoid redundancy and facilitate reading.
- **Response**: we merged them
- **Suggestion 3:** The column for the total tested in Table 2 is redundant to Table 1. I recommend removing it, as this information is already included in Table 1.
- **Response**: we delete it. Thank you

Discussion

5. **Comparison with other studies:** The comparison with other studies is made but could be expanded to include more details on why certain prevalences are higher or lower compared to other regions.

   Response: Thank you for your positive feedback. We have tried to expand on the comparable results by adding their justification.

- **Suggestion:** Include an explanation of why diarrhea lost significant association after adjustment in the multivariate model and cite relevant studies, as it is a significant predictor in undernutrition. I suggest citing the article "Predictors of nutritional recovery time in children aged 6–59 months with severe acute malnutrition in Sofala Province, Mozambique: survival analysis approach" by Audêncio Victor et al., published in the Journal of Public Health. This study can be accessed at: https://doi.org/10.1093/pubmed/fdae049.
- **Response**: we included the above article in the revised manuscript.

6. **Implications of the results:** The discussion addresses well the implications of the findings for public health. However, it would be useful to include more concrete suggestions on how interventions can be implemented in conflict areas.

   Response: thank you for your feedback; we have tried to include the above comment.

7. **Study limitations:** The limitations are mentioned but could be discussed in more detail, especially regarding potential selection biases and data collection limitations.

   Response: we included the limitation of the study in the revised document and indicted the limitations in the revised manuscript.

Conclusion

8. **Summarize the main findings:** The conclusion adequately summarizes the main findings of the study and suggests the need for integrated strategies to combat malaria and undernutrition in conflict regions.

   Response: thank you.

Structure and Clarity

9. **Manuscript Organization:** The manuscript is well-organized, but the introduction could be more concise.

   Response: In this revised manuscript, we have made the introduction section more organized.

10. **Language and Grammar:** The language is clear, but additional grammatical revision may be needed to ensure fluency.

    Response: We made grammatical revisions throughout the document to improve it.

11. Ethical Considerations, **Ethical Approval:** Obtaining ethical approval and informed consent is well documented, which is essential for publication.

Response: thank you.

Recommendations for the Authors

12. **Improvement in Methodology Description:** Include more details about the validation of the questionnaire and control of confounding variables.

    Response: thank you, we stated in the revised manuscript.

13. **Deeper discussion of limitations:** Expand the limitations section to include more details about possible biases and limitations of the study.

    Response: we expand the limitation of the study in the revised version

14. **Expansion of discussion on interventions:** Provide more concrete and detailed suggestions on how interventions can be implemented and evaluated in conflict contexts.

    Response: we have tried to make concrete. Thank you again.
